# Prognostic value of FOXP3+ regulatory T cells for patients with locally advanced oropharyngeal squamous cell carcinoma

**DOI:** 10.1371/journal.pone.0274830

**Published:** 2022-10-06

**Authors:** Joon Young Hur, Bo Mi Ku, Sehhoon Park, Hyun Ae Jung, Se-Hoon Lee, Myung-Ju Ahn

**Affiliations:** 1 Division of Hematology and Oncology, Department of Internal Medicine, Hanyang University Guri Hospital, Guri, Republic of Korea; 2 Research Institute for Future Medicine, Samsung Medical Center, Sungkyunkwan University School of Medicine, Seoul, Republic of Korea; 3 Division of Hematology-Oncology, Department of Medicine, Samsung Medical Center, Sungkyunkwan University School of Medicine, Seoul, Republic of Korea; The University of Burdwan, INDIA

## Abstract

**Background:**

Oropharyngeal squamous cell carcinoma (OPSCC) is the most common neoplasm originating at the base of the tongue or in the tonsils or soft palate. In this study, we investigated the prognostic value of FOXP3+ regulatory T cells in OPSCC.

**Methods:**

Tumor tissues of patients with locally advanced OPSCC were analyzed using quantitative multiplex immunohistochemistry. Staining of CD8+ T cells, conventional CD4+FOXP3- T cells (Tconv cells), CD4+FOXP3+ regulatory T cells (Treg cells), CD20+ B cells, and CD68+ macrophages was performed, and cell density was evaluated in both the tumor and its stroma.

**Results:**

Among the 71 patients included in this study, males constituted 93.0% of the cohort, and the median age was 59 years (range: 42–80 years). A total of 56 patients (78.9%) had a smoking history, and 53 (74.6%) patients were positive for human papillomavirus (HPV). The most frequent site of OPSCC was the tonsils (70.4%), followed by the base of the tongue (25.4%). The proportion of Treg cells was lower in the tumors of patients with HPV than in those of patients without HPV. Patients with OPSCC whose tumor Treg cell levels were above the median had longer relapse-free survival (RFS) periods than those with tumor Treg cell levels below the median (HR, 0.12; 95% CI, 0.03–0.46; p = 0.02). Our multivariate analysis identified high Treg levels (HR, 0.13; 95% CI, 0.02–1.00; p = 0.05) as an RFS factor that predicted a good prognosis.

**Conclusions:**

Our results demonstrated that high Treg cell density in locally advanced OPSCC tumors was correlated with longer RFS.

## Introduction

Oropharyngeal squamous cell carcinoma (OPSCC) is the most common neoplasm originating at the base of the tongue (BOT) and in tonsils, soft palate, or pharyngeal walls. OPSCCs are etiologically heterogeneous; some cases are attributed to sexually acquired human papillomavirus (HPV) infection, and others are attributed to tobacco and alcohol consumption [1]. Since the presence of immune cells is likely to affect the initiation, promotion, and progression of the tumor microenvironment (TME), the development of OPSCC can be, at least partly, attributed to the failure of the immune system to inhibit tumor progression and eliminate the developing tumor [2]. T lymphocytes are the primary immune cells involved; however, dendritic cells, B lymphocytes, plasma cells, various natural killer cells, and macrophages also contribute to the immune reaction. The absolute numbers of CD3+, CD4+, and CD8+ T cell subsets in patients with OPSCC were significantly lower than those in a cohort of healthy volunteers [3]. OPSCCs can induce apoptosis in CD8+ T cells via the mitochondrial and Fas/FasL pathways [4]. CD4+FOXP3+ regulatory T cells (Treg cells) can induce CD8+ T cell apoptosis and inhibit CD4+ T cell proliferation. In contrast, CD4+ T cells induce apoptosis of Treg cells, which, in turn, leads to the reduction of interleukin (IL)-2 levels [5]. The main immunohistochemical markers characterizing various types of T lymphocytes are CD4 for helper T cells, CD8 for cytotoxic T cells, and CD25 and fork head box p3 (FOXP3) for Treg cells [5]. Currently, multiplex immunohistochemistry (IHC) can provide quantitative data about tumor immune environments, including both the numbers of immune subgroups and their locations (tumor or stroma) [6]. Compared with conventional IHC, quantitative pathology enables single-cell quantitation of many biomarkers involved in cancer immune reactions in a single formalin-fixed paraffin-embedded (FFPE) tissue section. Unlike conventional IHC, which can detect only one marker per tissue sample, multiplex IHC can detect multiple markers from a single tissue sample and provide comprehensive information about the cell composition and spatial arrangement [7]. In this study, we used multiplex IHC to examine the distributions of immune cells in tumors and stroma and assessed the correlation between their characteristics and clinical features. We investigated the prognostic value of the markers detected in the intra-tumoral and stromal compartments of OPSCCs in comparison to other risk factors (e.g., HPV infection, the stage of the disease, and smoking history).

## Materials and methods

### Patients

Tumor tissues were obtained from patients with locally advanced OPSCC who were treated at Samsung Medical Center (SMC) in South Korea between October 2011 and December 2014, and all patients were followed up for at least five years. We obtained the specimens from the primary site. OPSCC staging was performed based on the seventh edition of the American Joint Committee on Cancer guidelines. All patients with locally advanced OPSCC had no distant metastasis. We retrospectively collected and reviewed follow-up data in the SMC cancer registry for adult patients with OPSCC. The selection criteria were histologically confirmed diagnosis of head and neck OPSCC and availability of clinical data from the beginning of therapy to the end of a follow-up duration of at least five years. We excluded patients diagnosed with adenoid cystic carcinoma, poorly differentiated carcinoma, adenosquamous carcinoma, or other types of head and neck cancer to ensure a study population that comprised only patients with OPSCC. Paraffin-embedded tumor samples were evaluated for HPV infection status using p16 IHC. To evaluate the profiles of various immune cells in both OPSCC tumors and their stroma, we quantified CD8+ T cell, conventional CD4+FOXP3- T cell (Tconv cell), CD4+FOXP3+ regulatory T cell (Treg cell), CD20+ B cell, and CD68+ macrophage populations using quantitative multiplex IHC of FFPE tissue specimens.

### Multiplex IHC (OPAL^TM^) staining

FFPE tissues were obtained as slices with a thickness of 4 μm, and multiplex IHC staining was performed using the following antibodies: CD8 (MCA1817T, Bio-Rad, Hercules, CA, dilution 1:300), CD4 (ab133616, Abcam, Cambridge, UK, dilution 1:100), CD20 (ab9475, Abcam, dilution 1:50), CD68 (M0876, DAKO, Carpinteria, CA, dilution 1:150), FOXP3 (ab20034, Abcam, dilution 1:100), and CK (pan antibody; AE-1/AE-3, NBP2-29429, NOVUS, Centennial, CO, dilution 1:500). Staining was repeated several times using the same experimental conditions. Slides were scanned using a Perkin-Elmer Vectra 3.0 Automated Quantitative Pathology Imaging System (Perkin-Elmer, Waltham, MA). Scanned images were analyzed using the Inform 2.2 and TIBCO Spotfire^TM^ (Perkin-Elmer) software. All immune cell populations from each panel were characterized and quantified using the cell segmentation tool in the InForm image analysis software. For co-expression analysis, the data obtained using the inForm software were exported to Spotfire™ software, and the threshold of positivity for each factor was determined based on IHC scoring methods. All cells in each slide were designated as positive or negative for each antibody, and the data were categorized and exported to a.xls file for analysis. We used the Spotfire™ program after the segmentation of tissues and cells, and the expression intensity was compared and examined based on the cut-off value. The numbers of CD8, FOXP3, CD68, CD20, CD4, and CK-positive cells were counted on each slide. After tumor cytokeratin (CK)-positive and stroma CK-negative areas were recognized, the proportion of each type of immune cell (CD8+ T cells, Tconv cells, Treg cells, CD20+ B cells, and CD68+ macrophages) was calculated as the number of positive-stained cells divided by the number of all nucleated cells (% positive cells/all nucleated cells) in the tumor and its stromal regions.

### Statistical analysis

Unpaired two-tailed Student’s t-testing was used to examine the significance of differences between continuous variables, and *p* values less than 0.05 were deemed to indicate significant differences. Relationships between paired data were analyzed using Pearson’s correlation coefficient (r). The χ2 test was used to examine differences in categorical variables. We examined the association between the proportion and localization of immune cells and both overall survival (OS) and relapse-free survival (RFS). OS was considered as the duration from the date of the beginning of treatment to the date of death or last follow-up, and RFS was considered as the duration from the date of the beginning of treatment to the date of cancer recurrence. We statistically tested the differences in survival using log-rank tests at α = 0.05. For the prognostic value of FOXP3+ Tregs, hazard ratios (HRs) and 95% confidence intervals (CIs) were adjusted for multiple prognostic factors using the Cox proportional hazards model. All statistical analyses were performed using GraphPad Prism Ver. 8 and IBM SPSS Statistics 27 (IBM Corp).

### Ethics statement

This study was approved by the Institutional Review Board of Samsung Medical Center (approval number: SMC 2018-02-009). Written informed consent was waived for this retrospective study.

## Results

### Patient characteristics

**Fig 1:**
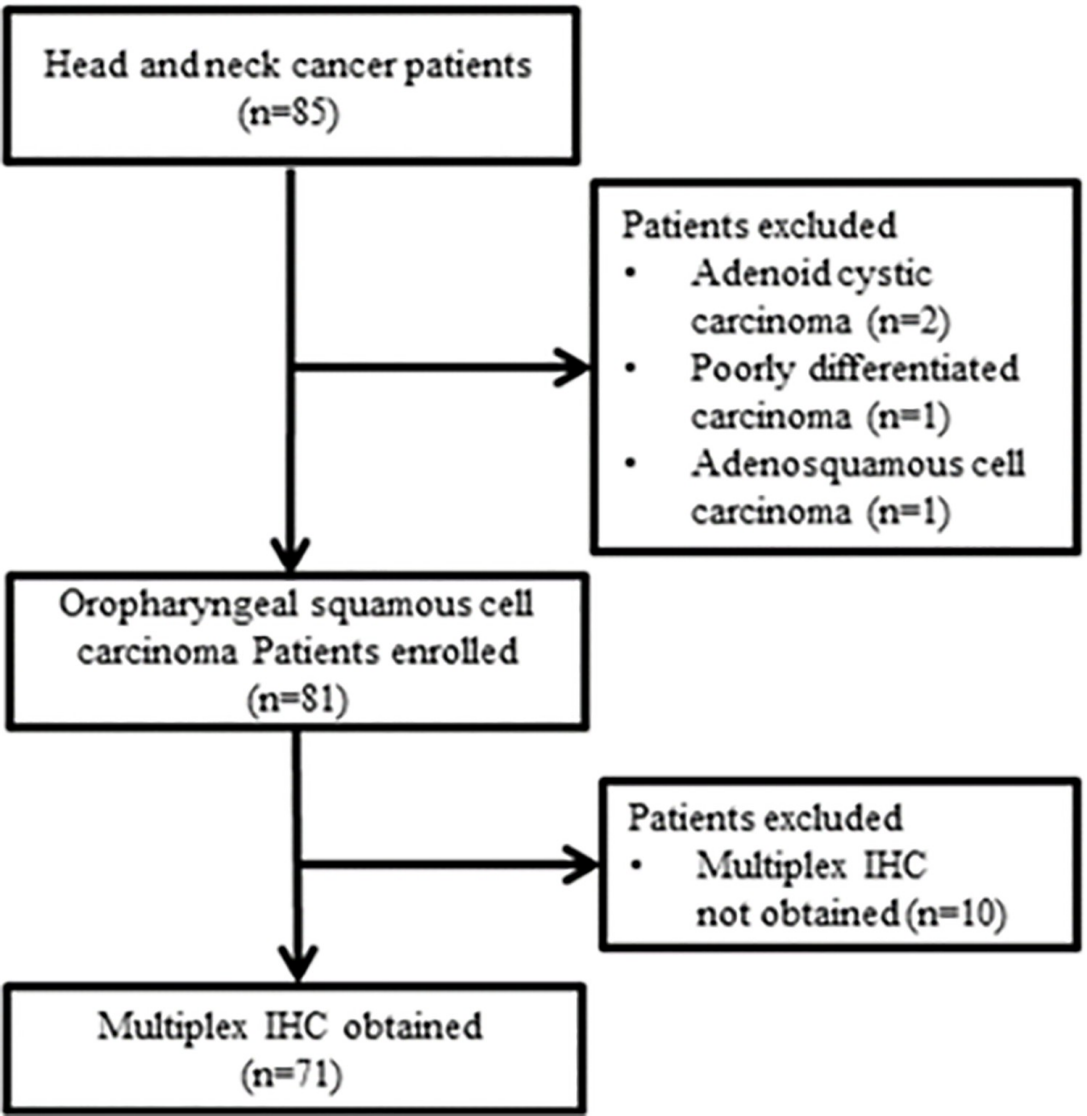
Consort diagram of the study methodology. Trial flow diagram of the study population. Among 85 patients with head and neck cancer, 81 patients with OPSCC were enrolled. Based on tissue availability, 10 patients were excluded from multiplexed-IHC analysis.

We identified 71 patients with OPSCC whose available pretreatment tumor tissue samples were considered adequate for multiplex IHC studies (Fig 1). The clinical characteristics of the patients are shown in Table 1. Among all patients, males constituted 93.0% of the cohort, and the median age was 59 years (range 42–80 years). A total of 56 patients (78.9%) had a history of smoking, and 53 patients (74.6%) were diagnosed positive for HPV. The most common origination site of head and neck OPSCC was the tonsils (70.4%), followed by the BOT (25.4%). A total of 33 (46.5%) received definitive concurrent chemoradiation therapy (CCRT), and 21 (29.6%) were treated with surgery, and adjuvant CCRT with cisplatin (100 mg/m^2^) was administered every three weeks. Among patients who received CCRT, twenty-three patients received cisplatin (100 mg/m^2^) every three weeks, eight received docetaxel (60 mg/m^2^) and cisplatin (60 mg/m^2^) every three weeks; and two patients received a loading dose of 400 mg/m^2^ of cetuximab followed by a weekly dose of 250 mg/m^2^.

**Table 1:** Baseline characteristics of patients with oropharyngeal squamous cell carcinoma.

Characteristics	All patients (n = 71)
Age (median, years) (range)	59 (42–80)
Sex, n (%)	
Female	5 (7.0%)
Male	66 (93.0%)
Smoking, n (%)	
Never smoker	15 (21.1%)
Ever smoker	56 (78.9%)
OPSCC diagnosis, n (%)	
Base of the tongue	18 (25.4%)
Tonsil	50 (70.4%)
Posterior pharyngeal wall	2 (2.8%)
Soft palate	1 (1.4%)
Stage, n (%)	
III	13 (18.5%)
IV	58 (81.5%)
HPV, n (%)	
Positive	53 (74.6%)
Negative	10 (14.1%)
Unknown	8 (11.3%)
Treatment, n (%)	
CCRT	33 (46.5%)
OP followed by CCRT	21 (29.6%)
Neoadjuvant CTx followed by CCRT	3 (4.2%)
OP alone	4 (5.6%)
OP followed by RTx	8 (11.3%)
RTx alone	2 (2.8%)

CCRT, concurrent chemoradiation therapy; CTx, chemotherapy; HPV, human papillomavirus; OP, operation; OPSCC, oropharyngeal squamous cell carcinoma; RTx, radiotherapy

### Heterogeneous distribution of immune cell subpopulations in OPSCC

**Fig 2:**
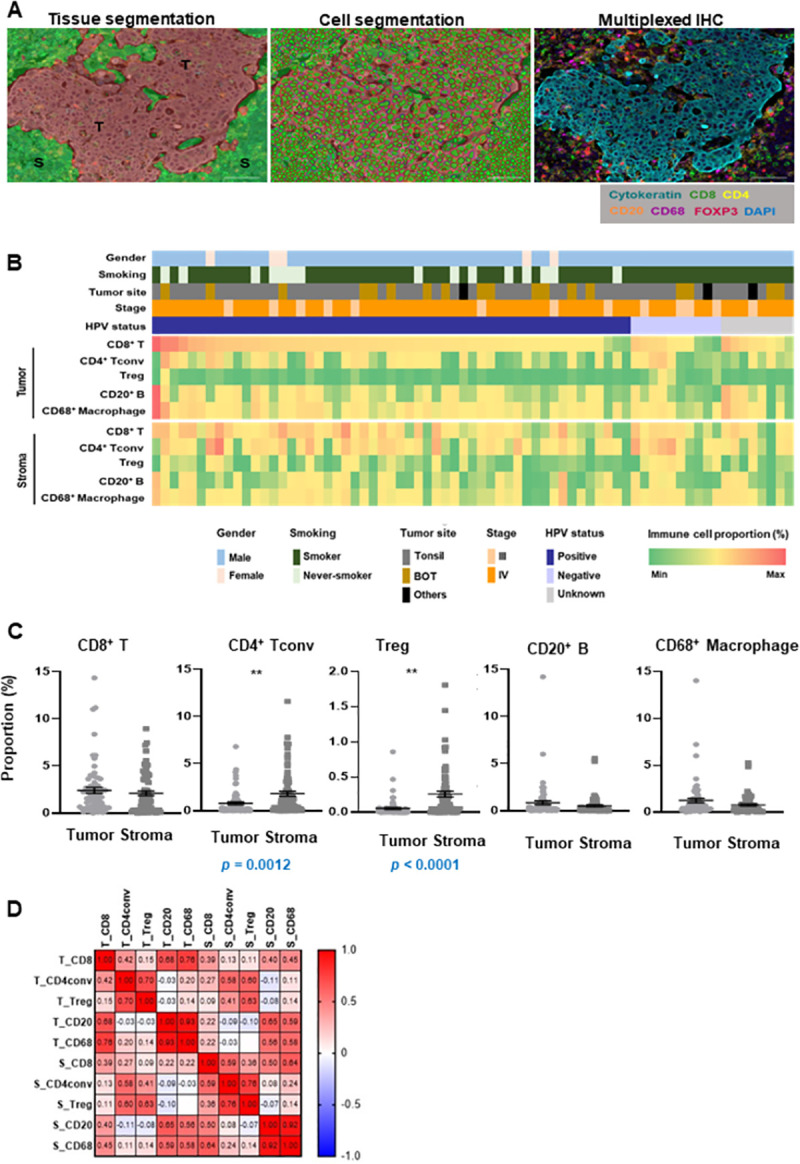
Immune profile of oropharyngeal squamous cell carcinoma (OPSCC). (A) Formalin-fixed paraffin-embedded (FFPE) sections of representative OPSCC tissues were analyzed by the panels of lineage-selective antibodies to identify lymphoid lineages. Representative multispectral images of OPSCC. Left panels; Tissue segmentation by InForm image analysis software (T: tumor; S: stroma). Middle panels; Cell segmentation using counterstaining with DAPI. Right panel; Multiplex image (cytokeratin: cyan, CD8: green, CD4: yellow, CD20: orange, CD68: magenta, FOXP3: red) (B) The landscape of patients with OPSCC. Upper panel: clinical characteristics, Lower panel: heatmap of immune cell proportion in 71 patients with OPSCC. Each column represents patient. (C) Immune cell distribution in the tumor and stroma. Data are represented as dot plots (bar: mean ± SE). P value (*p*) was calculated using two-sided Student’s t-test. (D) Pearson-correlation matrix of immune cells in the tumor and stromal regions.

The proportions of immune cell subsets (CD8+ T cells, Tconv cells, Treg cells, CD20+ B cells, and CD68+ macrophages) were analyzed in both tumors and stroma. The regions of interest (ROI) corresponding to the tumor and the stroma were analyzed. Approximately 70% of the total ROI area comprised tumor, regardless of the tissue type or the tumor stage (S1 Fig). As shown in Fig 2, OPSCC specimens showed various immune cell densities across tumors and stroma. CD8+ T cells were the most dominant immune cell population in OPSCC (Fig 2B). Tconv cells and Treg cells were more abundant in the stroma than in the tumor (Fig 2C). CD8+ T cell infiltration was significantly and positively correlated with the proportions of Tconv cells, Treg cells, CD20+ B cells, and CD 68+ macrophages in the tumor and stroma (Fig 2D).

### Association between HPV status and immune cell infiltration

**Fig 3:**
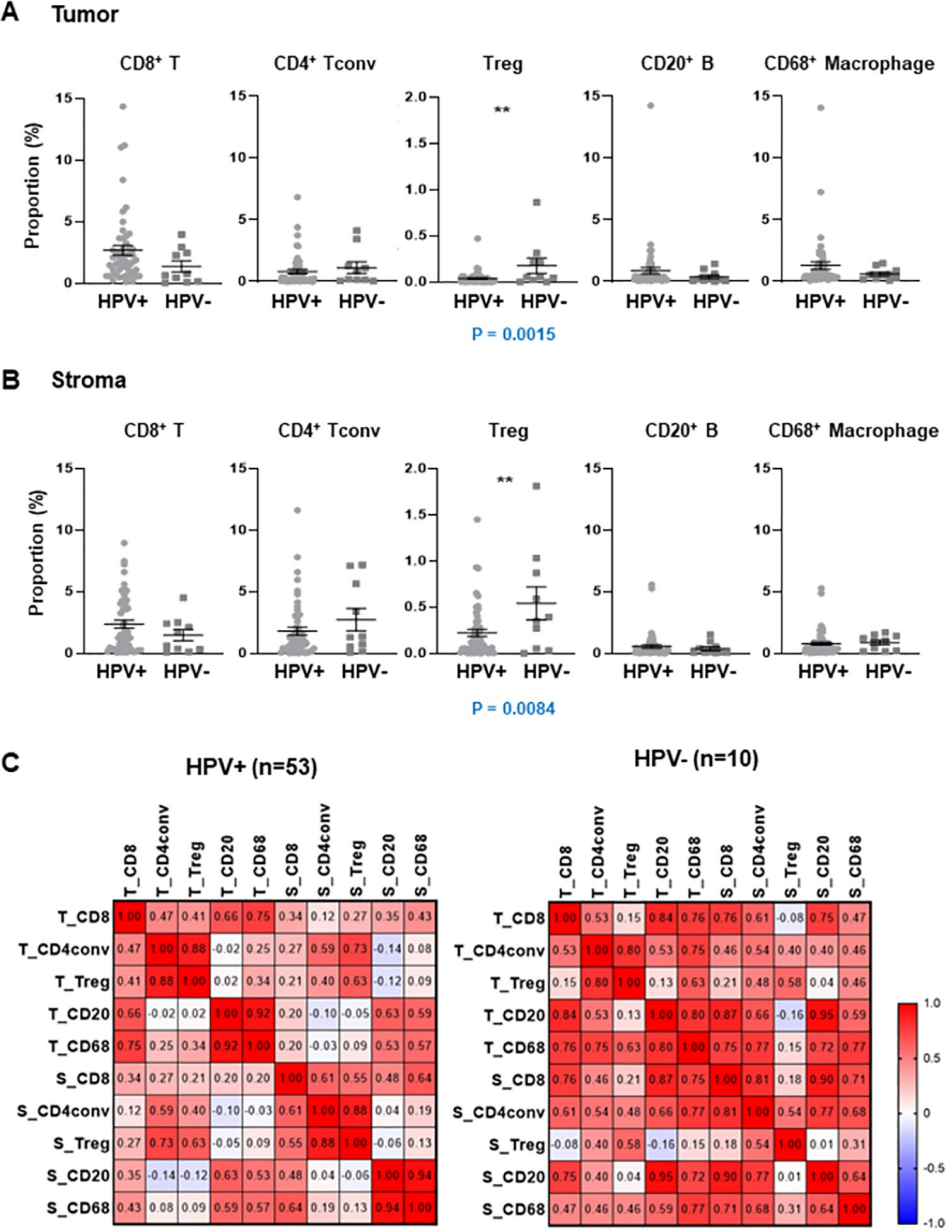
Immune cell proportion according to the HPV infection status. (A and B) Immune cell distribution according to p16 immunohistochemistry (IHC) analysis in the tumor region (A) and the stroma region (B). Data are represented as dot plots (bar: mean ± SE). P value (*p*) was calculated using two-sided Student’s t-test. (C) Pearson-correlation matrix of immune cells in the tumor and stromal regions according HPV status. HPV status was determined by p16 IHC. According to the results of the p16 IHC analysis, the distribution of other immune cells did not differ.

A total of 53 patients were diagnosed positive after p16 IHC analysis. No results were obtained for eight patients. When patients were classified by clinicopathological features, HPV status was not significantly associated with any clinical parameters (site of cancer, stage, smoking status, gender, or age). Since HPV status is a favorable prognostic marker for OPSCC, we investigated the correlation between HPV infection status and immune cell density. The proportion of Treg cells in the tumor was lower in patients with HPV than in patients diagnosed negative for HPV infection (Fig 3A). The proportion of Treg cells in the stroma also was lower in patients with HPV than in patients diagnosed negative for HPV infection (Fig 3B). The distribution of other immune cells did not differ according to the HPV infection status (Fig 3C).

### Immune cell distribution between tonsillar and BOT OPSCC

**Fig 4:**
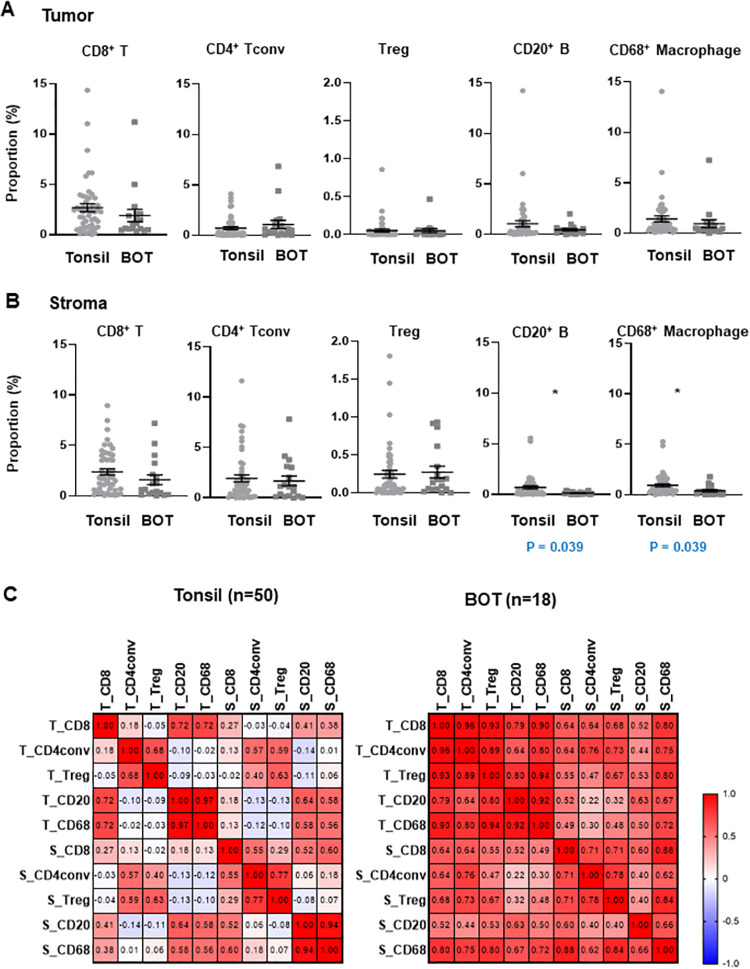
Immune cell distribution between tonsillar and BOT OPSCC. (A and B) Immune cell distribution according to the site of OPSCC (tonsil vs. BOT) in the tumor region (A) and the stromal region (B). Data are represented as dot plots (bar: mean ± SE). P value (*p*) was calculated using two-sided Student’s t-test. (C) Pearson-correlation matrix of immune cells in the tumor and stromal regions according the site of OPSCC (tonsil vs. BOT). The distribution of immune cells differed between tonsillar and BOT OPSCC.

The proportion of CD20+ B cells in the tumor stroma was higher in patients with tonsillar OPSCC than in patients with BOT OPSCC (p = 0.039, Fig 4A). The proportion of CD 68+ macrophages in the stroma was also higher in patients with tonsillar OPSCC than in patients with BOT OPSCC (p = 0.039, Fig 4B). The distribution of immune cells differed between tonsillar and BOT OPSCC (Fig 4C). The distribution of immune cells in the tumor and stroma did not differ significantly according to the site of OPSCC.

### Proportion of Treg cells in the tumor was associated with longer relapse-free survival

**Fig 5:**
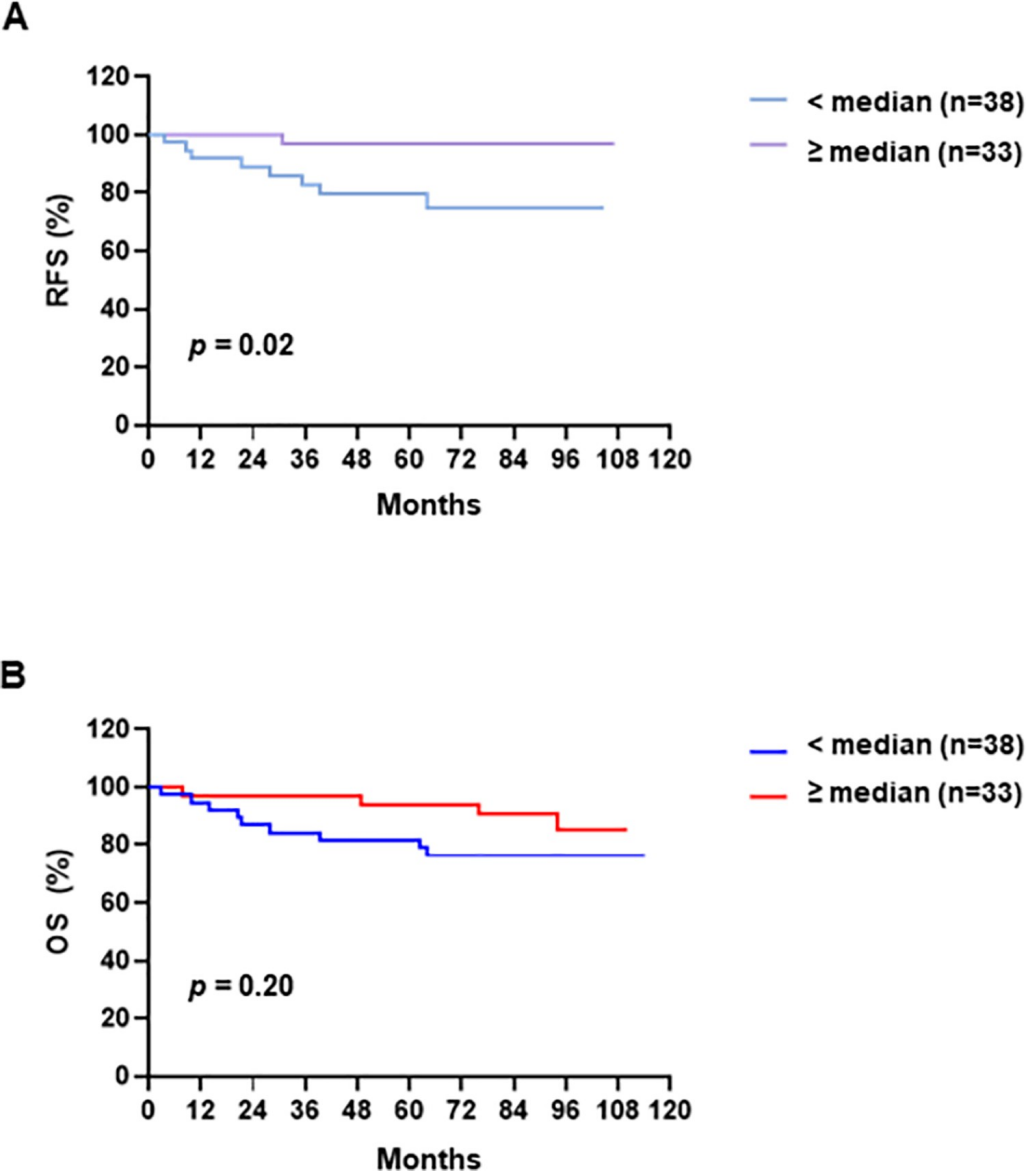
Kaplan-Meier analyses of OPSCC stratified by Treg proportion in the tumor region. (A) Relapse-free survival (RFS) and (B) overall survival (OS) in patients with OPSCC stratified by Treg cells in the tumor region. RFS and OS in relation to low (< median) or high frequencies (≥ median) of Treg cells were plotted by Kaplan-Meier survival curves. Statistical significance was determined by log-rank (Mantel-Cox) regression analysis.

Patients with OPSCC demonstrating Treg cell levels above the median in the tumor had longer RFS than those with a below-median level of Treg cells in the tumor (HR, 0.12; 95% CI, 0.03–0.46; p = 0.02) (Fig 5A). After adjusting for other known predictors, the multivariate analysis identified that a high Treg level (HR, 0.13; 95% CI, 0.02–1.00; *p* = 0.05) was associated with longer RFS (Table 2). However, CD20+ B cells, CD68+ macrophages, Tconv cells, and the ratio of CD8+ T cells to Treg cells did not correlate with any significant difference in RFS. The OS in patients with an above-median level of Treg cells in the tumor was longer than that of patients with a below-median level of Treg cells in the tumor; however, this difference was not significant (p = 0.20) (Fig 5B). The proportions of CD20+ B cells, CD68+ macrophages, and Tconv cells, and the ratio of CD8+ T cells to Treg cells did not differ significantly according to the OS (S2 Fig). Furthermore, no immune cell proportions or clinical characteristics were correlated with significant differences in the OS (S1–S4 Tables, S3 Fig).

**Table 2:** Univariate and multivariate analyses of prognostic factors for survival.

RFS	Univariate	Multivariate		
	*p*-value	HR	95% CI	*p*-value
Smoking: Ever smoker vs. Never smoker	0.10	Undefined		
Stage: IV vs. III	0.59	1.76	0.22–14.10	0.59
Treg: ≥ Median vs. < Median	0.02	0.13	0.02–1.00	0.05
HPV: Positive vs. Negative	0.40	0.47	0.09–2.46	0.37
OS	Univariate	Multivariate		
	*p*-value	HR	95% CI	*p*-value
Smoking: Ever smoker vs. Never smoker	0.20	3.49	0.45–26.89	0.22
Stage: III vs. IV	0.82	1.18	0.26–5.35	0.82
Treg: ≥ Median vs. < Median	0.20	0.47	0.14–1.53	0.21
HPV: Positive vs. Negative	0.58	0.39	0.10–1.51	0.17

CI, confidence interval; HR, hazard ratio; HPV, human papillomavirus

## Discussion

In this study, quantitative multiplex IHC revealed various immune cell densities across OPSCC tumor and stromal regions. Although CD8+ T cells were the most dominant immune cells and correlated significantly with infiltration of Tconv cells, Treg cells, CD20+ B cells, and CD 68+ macrophages, the distribution of various immune cells was heterogeneous. Of note, RFS was significantly longer in patients with an above-median proportion of Treg cells than in patients with a below-median proportion of Treg cells. This result is consistent with those of the previous studies [8, 9]. In contrast, another study reported that patients with strong CD8 expression had significantly better clinical outcomes than patients with low infiltration of tumor infiltrating lymphocytes (TIL) [10].

Treg cells are a subset of T cells that express the IL-2 receptor and FOXP3 flanking helix nuclear transcription factor. Treg cells play a major role in maintaining immune homeostasis and preventing autoimmune diseases [11]. Treg cells are typically considered immunosuppressive and have been linked to poor outcomes in several types of solid tumors, including ovarian, pancreatic, and hepatocellular carcinomas [12]. In contrast, a large proportion of Treg cells is a positive prognostic marker in patients with OPSCC [13]. Therefore, further studies are needed to elucidate whether the function of Treg cells differs according to the tumor type.

HPV-positive OPSCC is frequently associated with an increase in the number of cytotoxic T lymphocytes and an increased ratio of CD8+/Treg cells [14]. However, we did not observe any correlation between immune cell density and p16 status in this study. In another study, a high Treg cell count in the stromal compartment was associated with improved prognosis, whereas it was associated with worsened prognosis when observed in the intraepithelial compartment [15]. Stromal cells in the TME interact closely with tumor cells and affect tumor cell behavior in various ways [16]. When patients were classified based on the tumor stage, stromal immune cells were more important in stage I–III patients, and intraepithelial immune cells appeared to be more important in stage IV patients [17].

p16, which is diagnosed via IHC, is a widely available surrogate biomarker demonstrating good agreement with HPV status, as determined by the gold standard HPVE6/E7 mRNA test [18]. In a previous study examining head and neck squamous cell carcinoma, improvement in the survival was observed with an increase in the TIL level in HPV-positive patients [19]. In other words, HPV positive OPSCC have a significantly better prognosis than HPV negative tumors [20]. Inconsistent with several other studies, HPV status did not affect clinical outcomes in our study. The lack of a statistical difference in survival based on p16 status in this study is likely due to small number of patients with p16 (HPV) negative disease.

Previous studies have demonstrated inconsistent results with respect to TIL density in HPV positive and HPV negative OPSCC [21]. In fact, HPV-positive squamous cell carcinoma showed a slightly lower proportion of Tregs than HPV-negative squamous cell carcinoma [22]. In contrast, the infiltration of FOXP3+ Tregs did not differ between HPV positive and HPV negative squamous cell carcinoma [23]. Another study demonstrated that irrespective of p16 and HPV status, higher FOXP3+ T cell count was a strong prognostic factor independent of classical risk factors, such as tobacco and alcohol use [24]. Therefore, further investigations of the relationship between immune cell infiltration and an HPV-specific immune response in OPSCC are warranted.

In our study, relatively high percentages of patients were expected to be alive at the end of the five-year follow-up period, and low percentages of patients were expected to show recurrence. Given that recurrences and deaths are considered important factors during the follow-up, regardless of treatment modalities, strict surveillance after treatment is important. It is worth noting that all patients were followed up for at least five years. Moreover, no patients were diagnosed at an early stage, and all patients with locally advanced OPSCC had no distant metastasis. Even with 81.5% of the patients diagnosed at stage four, the survival rate was high in this study.

This study has some limitations. First, the retrospective nature of the study and limited sample size might have biased our interpretation of the data. Also, considering that most included patients had favorable prognoses, which may have contributed to sample selection bias. Second, since the patients in this study did not receive immunotherapy, the predictive value of our analysis could not be evaluated. Third, other immune cell biomarkers, such as CD25, CD45, CD56, CD127, and programmed death-ligand 1 (PD-L1), were not analyzed in this study. Finally, additional HPV DNA polymerase chain reaction (PCR) data were not obtained in this study. While p16 positivity shows high sensitivity for the detection of HPV associated OPSCC, the combination of p16 IHC and HPV PCR with a further study thus appears advisable.

## Conclusions

Our results demonstrated that a large proportion of Treg cells in the tumor was correlated with longer RFS in patients with locally advanced OPSCC and might have predictive value. Further studies are needed to evaluate whether the proportion of tumor Treg cells can predict clinical outcomes of patients with OPSCC treated with immune checkpoint inhibitors.

## Supporting information

S1 Table Relapse-free survival according to immune cell proportions. (DOCX)

S2 Table Overall survival according to immune cell proportions. (DOCX)

S3 Table Relapse-free survival according to clinical characteristics. (DOCX)

S4 Table Overall survival according to clinical characteristics. (DOCX)

S1 Fig Tumor and stromal proportion in tissue. The proportion of tumor and stromal region of FFPE samples were indicated according to the site of OPSCC (BOT, tonsil, and others) or stage (III and IV). Data are represented as dot plots (bar: mean ± SE). P value (*p*) was calculated using one-way ANOVA or two-sided Student’s t-test.
(TIF)

S2 Fig Kaplan-Meier analyses of OPSCC stratified by the proportion of CD8/Treg cells in the tumor region. (A) Relapse-free survival (RFS) and (B) overall survival (OS) in patients with OPSCC stratified by CD8/Treg ratio in the tumor region. RFS and OS in relation to low (< median) or high ratio (≥ median) of CD8/Treg were plotted by Kaplan-Meier survival curves. Statistical significance was determined by log-rank (Mantel-Cox) regression analysis.
(TIF)

S3 Fig Kaplan-Meier analyses of OPSCC stratified by the proportion of Treg cells in the stromal region. (A) Relapse-free survival (RFS) and (B) overall survival (OS) in patients with OPSCC stratified by Treg cells in the stroma region. RFS and OS in relation to low (< median) or high frequencies (≥ median) of Treg cells were plotted by Kaplan-Meier survival curves. Statistical significance was determined by log-rank (Mantel-Cox) regression analysis.
(TIF)
