# Supplementary material for: Prognostic value of FOXP3+ regulatory T cells for patients with locally advanced oropharyngeal squamous cell carcinoma
Source: PLoS One. 2022 Oct 6;17(10):e0274830. doi: 10.1371/journal.pone.0274830 (PMC9536544; doi:10.1371/journal.pone.0274830)
Supplement: S1 Table — (DOCX) [file pone.0274830.s001.docx]

Supplementary Table 1. Relapse-free survival according to immune cell proportions

| Immune cell proportions in the OPSCC tumor | | No. of Events/  No. of Patients | p-value | HR (95% CI) |
| --- | --- | --- | --- | --- |
| CD68 | ≥ Median | 6/35 | 0.30 | 2.06 (0.56 – 7.59) |
|  | < Median | 3/36 |  | 0.49 (0.13 – 1.80) |
| CD20 | ≥ Median | 5/35 | 0.78 | 1.21 (0.33 – 4.45) |
|  | < Median | 4/36 |  | 0.83 (0.22 – 3.07) |
| Treg | ≥ Median | 1/33 | 0.02 * | 0.13 (0.03 – 0.47) |
|  | < Median | 8/38 |  | 7.91 (2.14 – 29.23) |
| Tconv | ≥ Median | 2/36 | 0.07 | 0.26 (0.07 – 0.96) |
|  | < Median | 7/35 |  | 3.85 (1.04 – 14.23) |
| CD8 | ≥ Median | 4/36 | 0.54 | 0.66 (0.18 – 2.47) |
|  | < Median | 5/35 |  | 1.50 (0.41 – 5.59) |

CI, confidence interval; HR, hazard ratio; OPSCC, oropharyngeal squamous cell carcinoma
