# Supplementary material for: Prognostic value of FOXP3+ regulatory T cells for patients with locally advanced oropharyngeal squamous cell carcinoma
Source: PLoS One. 2022 Oct 6;17(10):e0274830. doi: 10.1371/journal.pone.0274830 (PMC9536544; doi:10.1371/journal.pone.0274830)
Supplement: S2 Table — (DOCX) [file pone.0274830.s002.docx]

Supplementary Table 2. Overall survival according to immune cell proportions

| Immune cell proportion in OPSCC tumor | | No. of Events/  No. of Patients | p-value | HR (95% CI) |
| --- | --- | --- | --- | --- |
| CD68 | ≥ Median | 7/35 | 0.69 | 1.24 (0.42 – 3.69) |
|  | < Median | 6/36 |  | 0.80 (0.27 – 2.39) |
| CD20 | ≥ Median | 6/35 | 0.71 | 0.81 (0.27 – 2.41) |
|  | < Median | 7/36 |  | 1.23 (041 – 3.65) |
| Treg | ≥ Median | 4/33 | 0.20 | 0.47 (0.16 – 1.41) |
|  | < Median | 9/38 |  | 2.11 (0.71 – 6.27) |
| CD4Tconv | ≥ Median | 6/36 | 0.72 | 0.82 (0.28 – 2.43) |
|  | < Median | 7/35 |  | 1.22 (0.41 – 3.63) |
| CD8 | ≥ Median | 7/36 | 0.87 | 1.10 (0.37 – 3.26) |
|  | < Median | 6/35 |  | 0.91 (0.31 – 2.70) |

CI, confidence interval; HR, hazard ratio; OPSCC, oropharyngeal squamous cell carcinoma
