# Supplementary material for: Prognostic value of FOXP3+ regulatory T cells for patients with locally advanced oropharyngeal squamous cell carcinoma
Source: PLoS One. 2022 Oct 6;17(10):e0274830. doi: 10.1371/journal.pone.0274830 (PMC9536544; doi:10.1371/journal.pone.0274830)
Supplement: S3 Table — (DOCX) [file pone.0274830.s003.docx]

Supplementary Table 3. Relapse-free survival according to clinical characteristics

| Baseline Factors | | No. of Events/  No. of Patients | p-value | HR (95% CI) |
| --- | --- | --- | --- | --- |
| Tissue | BOT | 2/18 | 0.81 | 0.83 (0.19 – 3.69) |
| (n=68) | Tonsil | 7/50 |  | 1.21 (0.27 – 5.39) |
| HPV status | Positive | 5/53 | 0.40 | 0.50 (0.07 – 3.65) |
| (n=63) | Negative | 2/10 |  | 2.00 (0.27 – 14.59) |
| Stage | IV | 8/58 | 0.59 | 1.76 (0.32 – 9.63) |
| (n=71) | III | 1/13 |  | 0.57 (0.10 – 3.11) |
| Smoking | Ever | 9/56 | 0.10 | Undefined |
| (n=71) | Never | 0/15 |  | Undefined |
| Gender | Female | 0/5 | 0.37 | Undefined |
| (n=71) | Male | 9/66 |  | Undefined |
| Age (years) | ≥ 59 | 5/37 | 0.81 | 1.17 (0.32 - 4.33) |
| (n=71) | < 59 | 4/34 |  | 0.85 (0.23 - 3.16) |

BOT, base of the tongue; CI, confidence interval; HPV, human papillomavirus; HR, hazard ratio
