# Supplementary material for: Prognostic value of FOXP3+ regulatory T cells for patients with locally advanced oropharyngeal squamous cell carcinoma
Source: PLoS One. 2022 Oct 6;17(10):e0274830. doi: 10.1371/journal.pone.0274830 (PMC9536544; doi:10.1371/journal.pone.0274830)
Supplement: S4 Table — (DOCX) [file pone.0274830.s004.docx]

Supplementary Table 4. Overall survival according to clinical characteristics

| Baseline Factors | | No. of Events/  No. of Patients | p-value | HR (95% CI) |
| --- | --- | --- | --- | --- |
| Tissue | BOT | 4/18 | 0.60 | 1.38 (0.38 – 4.98) |
| (n=68) | Tonsil | 8/50 |  | 0.72 (0.20 – 2.61) |
| HPV status | Positive | 7/53 | 0.58 | 0.65 (0.11 – 3.92) |
| (n=63) | Negative | 2/10 |  | 1.55 (0.26 – 9.37) |
| Stage | IV | 11/58 | 0.82 | 1.19 (0.29 – 4.92) |
| (n=71) | III | 2/13 |  | 0.84 (0.20 – 3.50) |
| Smoking | Ever | 12/56 | 0.20 | 3.50 (0.95 – 12.83) |
| (n=71) | Never | 1/15 |  | 0.29 (0.08 – 1.05) |
| Gender | Female | 1/5 | 0.94 | 1.09 (0.13 – 8.99) |
| (n=71) | Male | 12/66 |  | 0.92 (0.11 – 7.62) |
| Age (years) | ≥ 59 | 9/37 | 0.19 | 2.16 (0.73 – 6.41) |
| (n=71) | < 59 | 4/34 |  | 0.46 (0.16 – 1.37) |

BOT, base of the tongue; CI, confidence interval; HPV, human papillomavirus; HR, hazard ratio
