# Supplementary figures and images for: Prognostic value of FOXP3+ regulatory T cells for patients with locally advanced oropharyngeal squamous cell carcinoma
Source: PLoS One. 2022 Oct 6;17(10):e0274830. doi: 10.1371/journal.pone.0274830 (PMC9536544; doi:10.1371/journal.pone.0274830)

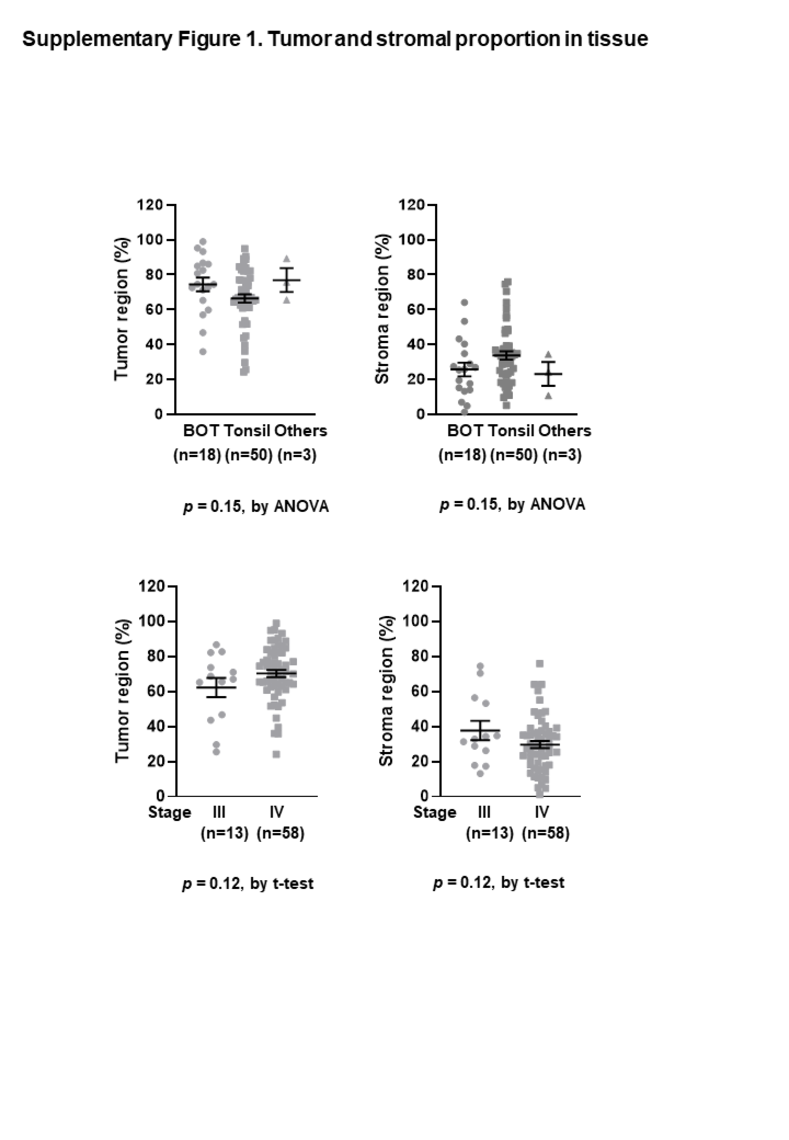

Supplement: S1 Fig — The proportion of tumor and stromal region of FFPE samples were indicated according to the site of OPSCC (BOT, tonsil, and others) or stage (III and IV). Data are represented as dot plots (bar: mean ± SE). P value (p) was calculated using one-way ANOVA or two-sided Student’s t-test. (TIF) [file pone.0274830.s005.tif]

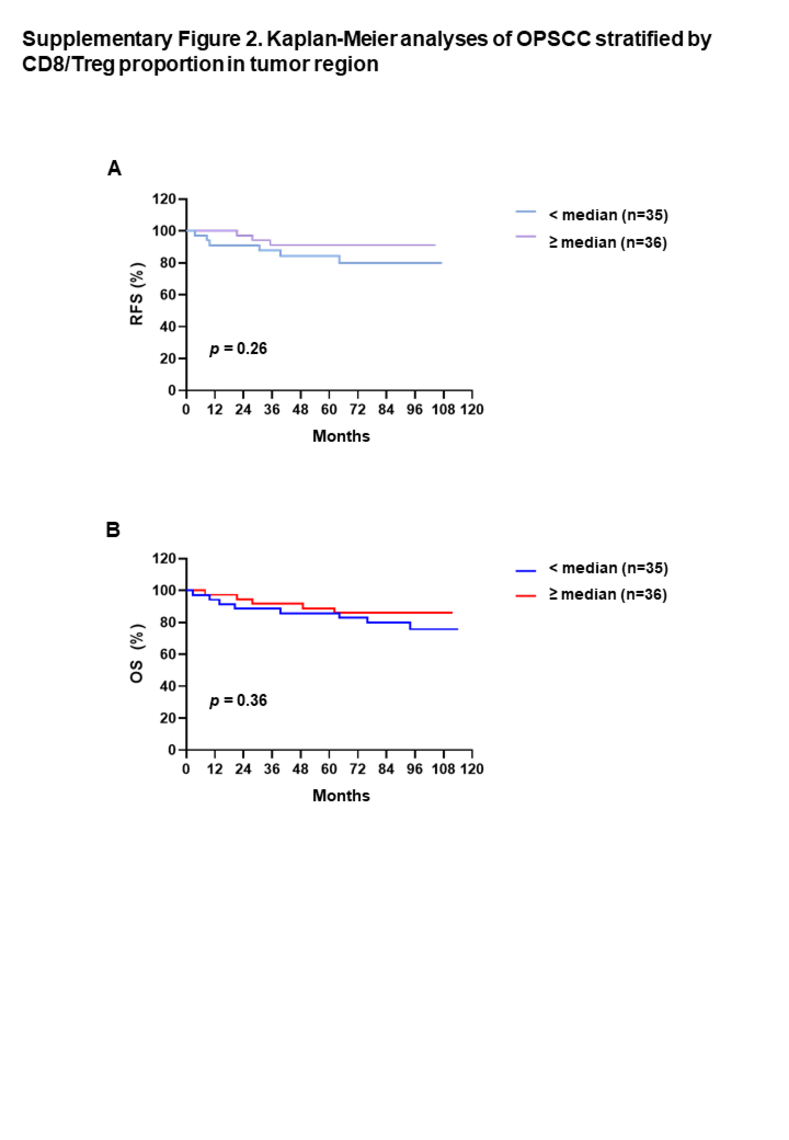

Supplement: S2 Fig — (A) Relapse-free survival (RFS) and (B) overall survival (OS) in patients with OPSCC stratified by CD8/Treg ratio in the tumor region. RFS and OS in relation to low (< median) or high ratio (≥ median) of CD8/Treg were plotted by Kaplan-Meier survival curves. Statistical significance was determined by log-rank (Mantel-Cox) regression analysis. (TIF) [file pone.0274830.s006.tif]

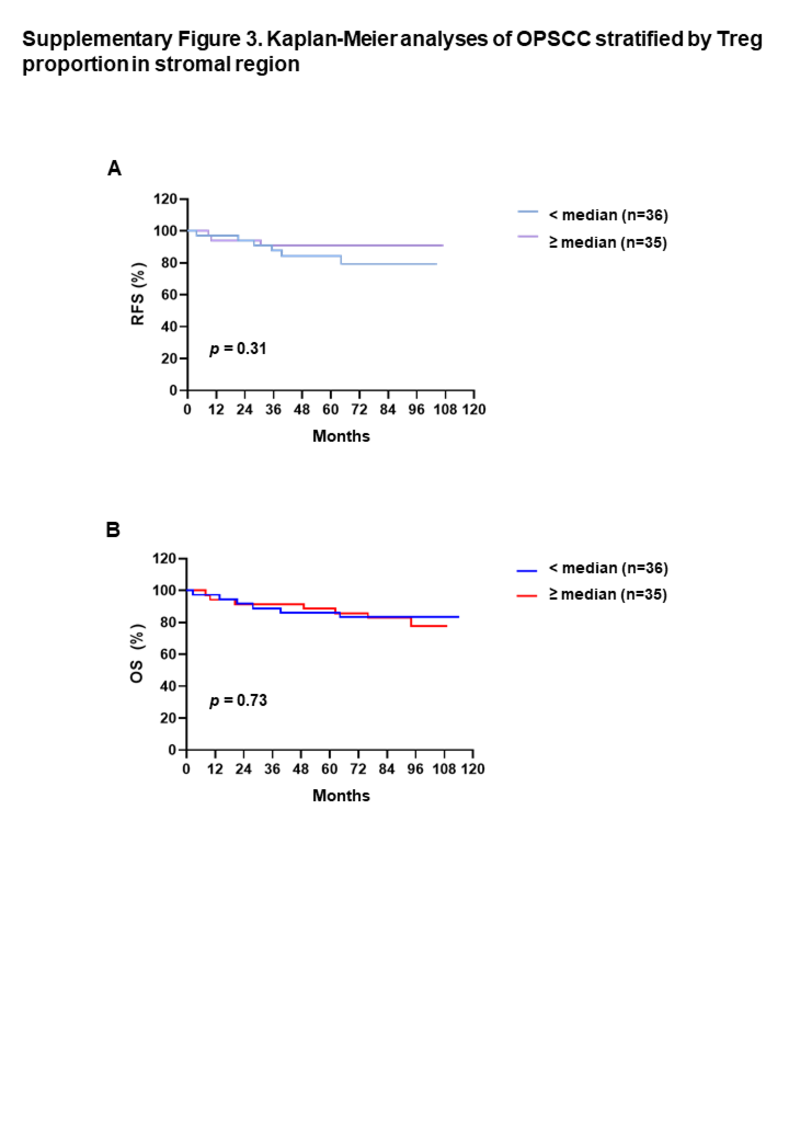

Supplement: S3 Fig — (A) Relapse-free survival (RFS) and (B) overall survival (OS) in patients with OPSCC stratified by Treg cells in the stroma region. RFS and OS in relation to low (< median) or high frequencies (≥ median) of Treg cells were plotted by Kaplan-Meier survival curves. Statistical significance was determined by log-rank (Mantel-Cox) regression analysis. (TIF) [file pone.0274830.s007.tif]
